# Identification of a shared, common haplotype segregating with an *SGCB* c.544 T > G mutation in Indian patients affected with sarcoglycanopathy

**DOI:** 10.1038/s41598-023-41487-6

**Published:** 2023-09-12

**Authors:** Shamita Sanga, Sudipta Chakraborty, Mainak Bardhan, Kiran Polavarapu, Veeramani Preethish Kumar, Chandrika Bhattacharya, Saraswati Nashi, Seena Vengalil, Thenral S. Geetha, Vedam Ramprasad, Atchayaram Nalini, Analabha Basu, Moulinath Acharya

**Affiliations:** 1 National Institute of Biomedical Genomics, P.O: N.S.S, Kalyani, West Bengal 741251 India; 2 Regional Centre for Biotechnology, Faridabad, India; 3 National Institute of Mental Health and Neurosciences, Bangalore, India; 4 Medgenome, MedgenomeLabs, Bommasandra, Bangalore, India

**Keywords:** Computational biology and bioinformatics, Genetics, Diseases, Medical research

## Abstract

Sarcoglycanopathy is the most frequent form of autosomal recessive limb-girdle muscular dystrophies caused by mutations in *SGCB* gene encoding beta-sarcoglycan proteins. In this study, we describe a shared, common haplotype co-segregating in 14 sarcoglycanopathy cases from 13 unrelated families from south Indian region with the likely pathogenic homozygous mutation c.544 T > G (p.Thr182Pro) in *SGCB*. Haplotype was reconstructed based on 10 polymorphic markers surrounding the c.544 T > G mutation in the cases and related family members as well as 150 unrelated controls from Indian populations using PLINK1.9. We identified haplotype H1 = G, A, G, T, G, G, A, C, T, G, T at a significantly higher frequency in cases compared to related controls and unrelated control Indian population. Upon segregation analysis within the family pedigrees, H1 is observed to co-segregate with c.544 T > G in a homozygous state in all the pedigrees of cases except one indicating a probable event of founder effect. Furthermore, Identical-by-descent and inbreeding coefficient analysis revealed relatedness among 33 new pairs of seemingly unrelated individuals from sarcoglycanopathy cohort and a higher proportion of homozygous markers, thereby indicating common ancestry. Since all these patients are from the south Indian region, we suggest this region to be a primary target of mutation screening in patients diagnosed with sarcoglycanopathy.

## Introduction

Limb girdle muscular dystrophies (LGMD) are heterogeneous group of disorders leading to progressive muscle wasting and weakness, predominantly characterized by limb girdle weakness^1^. It is caused by mutations in 32 genes causing different type of LGMDs^2^. Sarcoglycanopathies (SG) are the most frequent form of autosomal recessive LGMD comprising of four subtypes- LGMDR3, LGMDR4, LGMDR5 and LGMDR6 caused by mutations in *SGCA*, *SGCB*, *SGCG* and *SGCD* encoding for the alpha-, beta-, gamma- and delta- sarcoglycan proteins respectively^3^. Sarcoglycans are tightly bound to each other and form a transmembrane glycoprotein across the cell membrane of skeletal and cardiac muscle fibres^4^. Mutation occurring in one gene may result in partial or total deficiency of all the other sarcoglycan protein in the complex thereby leading to the loss of muscle membrane integrity^5^.

In recent years, genetic analysis of LGMDs have increasingly been undertaken in various parts of India. Srinivas et al.^6^ presented their initial observations on 35 patients with LGMDs. In the following four decades, several studies have accumulated describing clinical phenotypes of LGMDs and diagnosis achieved by immunohistochemistry. Khadilkar et al.^7^ analysed the genetic aspects of 18 SG patients from India. Among them, *SGCG* gene mutation (44.4%) was most common followed by *SGCD* (27.77%), *SGCA* (22.22%), and *SGCB* (5.55%). Among *SGCG* mutation, 525delT was encountered in 50% of the cases although haplotype analysis has not been undertaken. Recently, Bardhan et al.^8^ investigated the clinical picture, genetic basis, and disease progression of 68 patients genetically confirmed to have SG. The most common mutation identified was c.544 T > G in *SGCB* detected in 20 patients (29.42%) from the southern states of Tamil Nadu, Karnataka and Andhra Pradesh in India indicating a founder event leading to the descend of this mutation from a common ancestor.

Mutations that descend from founder events are typically inherited as part of a haplotype. By making use of tens of thousands of single nucleotide polymorphisms (SNPs) extracted from whole-genome sequencing data, fine scale resolution of shared ancestral haplotypes can be identified. Genomic regions that have been inherited from a recent common ancestor, said to be identical by descent (IBD) can also be identified. Such methods have been useful in many applications, including disease mapping^9,10^ and uncovering unknown relatedness^11,12^. Individuals who inherit part of a founder haplotype are in fact IBD over this genomic region. Inferring such IBD regions and uncover unknown relatedness can be useful in identifying founder events which will be beneficial in genetically informed risk stratification and management of prevention of SG.

In this study, we describe the inheritance of likely pathogenic homozygous mutation c.544 T > G in *SGCB* in 14 genetically and clinically confirmed SG cases from 13 unrelated families reported by Bardhan et al.^8^ as part of a common 10-SNP haplotype. This haplotype on chromosome 4 outlines a genomic fragment of approximately 238 kb and is observed to be present significantly at higher frequency in the SG cases and related controls compared to 150 unrelated controls of general Indian population. We also performed an IBD analysis leveraging sequencing data to investigate relatedness between the families and visualise clusters of individuals sharing IBD and draw conclusions as to the presence of common ancestor. Additionally, we have also performed inbreeding coefficient (F-value) analysis of SNP markers from sequence data in the SG cases and related controls as well as unrelated controls of Indian population. The median estimated F-value is calculated based on the observed versus expected number of homozygous genotypes and its mean distribution is plotted. Such an analysis would provide a measure of the proportion by which the heterozygosity of an individual is reduced by inbreeding, indicating that two alleles at any given locus are identical by descent from the common ancestor(s) of the two parents. It provides information on relatedness among parents, population structure and recent demographic events^13^.

## Materials and methods

All methods were performed in accordance with the relevant guidelines and regulations^14,15^.

### Sample cohort

Our study sample was composed of 27 individuals (14 genetically and clinically confirmed SG cases and 13 related family members) from 13 unrelated families originating from the southern states of Tamil Nadu, Karnataka and Andhra Pradesh in India. The SG cases (including a pair of siblings) were affected and homozygous carrier of c.544 T > G mutation in *SGCB* gene, 13 related individuals were heterozygous carrier (Table 1). The patient’s clinical picture, genetic basis, and disease progression are reported by Bardhan et al.^8^. The clinical details of the cases are summarized in Fig. S1. The study was approved by the National Institute of Mental Health and Neurosciences ethics committee (NIMHANS/IEC/2020-21). Written informed consent was obtained from the parents and/or legal guardians of all participants in the study.

**Table 1:** SG sample cohort.

Pedigree #	Individual ID	*SGCB* mutation	Genotype
Ped1	SG-1	c.544 T > G	G/G
Ped2	SG-2	c.544 T > G	G/G
Ped3	SG-3	c.544 T > G	G/G
	SG-3-S	c.544 T > G	G/G
	SG-3-P1	c.544 T > G	G/T
Ped4	SG-4-P1	c.544 T > G	G/T
	SG-4-P2	c.544 T > G	G/T
	SG-4	c.544 T > G	G/G
Ped5	SG-5	c.544 T > G	G/G
Ped6	SG-6	c.544 T > G	G/G
	SG-6-P1	c.544 T > G	G/T
	SG-6-P2	c.544 T > G	G/T
Ped7	SG-7	c.544 T > G	G/G
	SG-7-P1	c.544 T > G	G/T
Ped8	SG-8	c.544 T > G	G/G
	SG-8-P1	c.544 T > G	G/T
	SG-8-P2	c.544 T > G	G/T
Ped9	SG-9	c.544 T > G	G/G
	SG-9-P1	c.544 T > G	G/T
	SG-9-P2	c.544 T > G	G/T
Ped10	SG-10	c.544 T > G	G/G
	SG-10-P1	c.544 T > G	G/T
	SG-10-P2	c.544 T > G	G/T
Ped11	SG-11	c.544 T > G	G/G
Ped12	SG-12	c.544 T > G	G/G
	SG-12-P1	c.544 T > G	G/T
Ped13	SG-13	c.544 T > G	G/G

*P1* parent 1, *P2* parent 2, *S* sibling.

Additionally, 150 unrelated Indian population controls were included in the study. While choosing our control group, we emphasized on diversity. The unrelated control population represents (1) different geographical regions (North, South, East); (2) different social hierarchies (Upper Caste, Lower Caste and Tribe) and (3) Different linguistic groups (Indo-Aryan, Dravidian speakers). 87 samples were from south Indian region from the populations: Iyengar (Chennai, Tamil Nadu; N = 6), Iyer (Chennai, Tamil Nadu; N = 13), Koya Dora (Andhra Pradesh; N = 11), Konda Dora (Visakhapatnam, Andhra Pradesh; N = 1), Konda Reddy (Andhra Pradesh; N = 17), Paniya (Kerala; N = 11), Toda (Nilgiri Hills; N = 20) and Kota (Nilgiri Hills; N = 8). 63 samples were from north Indian region from the populations: Rana Tharu (Uttar Pradesh; N = 12), West Bengal brahmins (Kolkata, West Bengal; N = 10), Tanti (Kolkata, West Bengal; N = 1), Saryuparin brahmin (Raipur, Chattisgarh; N = 14), Saurastra Brahmin (Maharashtra; N = 9), Khatri (Amritsar; N = 11) and Chamar (Punjab, Haryana; N = 6).

Genotypes for the SG cases and related controls were obtained from the variant call format (VCF) generated from the BAM files provided by Medgenome, Bangalore, India^8^ (Table S1). Genotypes of 150 unrelated Indian population controls were taken from the GenomeAsia 100 K database^16^ (Table S2).

### Homozygosity mapping and selection of neutral markers

The genotype data per sample were subjected for homozygosity mapping on Web based tool Homozygosity Mapper (http://www.homozygositymapper.org/)^14^. Ten single-nucleotide polymorphisms (SNPs) as polymorphic sites surrounding the c.544 T > G were selected as neutral markers for the haplotype reconstruction: rs10009426, rs6824707, rs6851073, rs2271046, rs225160, rs225170, rs999634, rs3860707, rs35414474 and rs17611952. These SNPs markers were chosen based on their chromosomal position and on their allelic frequencies. They are located on chromosome 4, in the *DCUN1D4*, *LRRC66*, *SGCB* and *SPATA18* genes, outlining a genomic fragment of approximately 238 kb from rs10009426 to rs17611952. The distance from rs10009426 to the c.544 T > G is 184060 bp and from rs17611952 to c.544 T > G is 54039 bp (Table 2).

**Table 2:** Selection of SNPs as neutral markers surrounding the mutant allele.

Gene	Nucleotide variants	SNP ID	Distance to *SGCB* c.544 T > G (bp)	Ancestral Allele	MAF (1KGP3-ALL)	MAF (1KGP3-SAS)	MAF (gnomAD-ALL)	MAF (gnomAD-SAS)
*DCUN1D4*	G/A	rs10009426	( −) 184,060	G	0.456 (A)	0.491 (A)	0.44 (G)	0.472 (G)
*DCUN1D4*	A/G	rs6824707	( −) 183,788	A	0.458 (G)	0.491 (G)	0.436 (A)	0.471 (A)
*DCUN1D4*	C/G	rs6851073	( −) 180,462	C	0.477 (C)	0.497 (C)	0.581 (C)	0.400 (C)
*DCUN1D4*	T/A	rs2271046	( −) 142,161	T	0.455 (A)	0.491 (A)	0.357 (T)	0.45 (T)
*LRRC66*	G/A	rs225160	( −) 10,734	G	0.644(G)	0.67(G)	0.563 (G)	0.648 (G)
*SGCB*	T/G	rs751427686	0	T	0 (G)	0 (G)	1.193e − 05 (G)	9.8e − 05 (G)
*SGCB*	A/G	rs225170	( +) 1077	A	0.644 (A)	0.673 (A)	0.564 (A)	0.648 (A)
*SPATA18*	C/A	rs999634	( +) 32,140	C	0.642 (C)	0.688(C)	NA	NA
*SPATA18*	T/C	rs3860707	( +) 43,270	T	0.616 (T)	0.661 (T)	0.508 (T)	0.605 (T)
*SPATA18*	C/G	rs35414474	( +) 53,861	C	0.158 (G)	0.299 (G)	0.127 (G)	0.277 (G)
*SPATA18*	A/T	rs17611952	( +) 54,039	A	0.157 (T)	0.3 (T)	0.125 (T)	0.276 (T)

### Haplotype analysis

Haplotype reconstructions based on the 10 markers selected including the c.544 T > G mutation for the two groups, cases and related controls, were conducted using PLINK1.9^17^ using expectation–maximization likelihood algorithm. An additional haplotype reconstruction based on the same 10 markers in the background of c.544 T > G mutation were conducted for the cases, related controls and 150 unrelated control groups from south and north Indian populations. The haplotype frequencies for cases, related and unrelated controls were estimated. Subsequently, the chi-square test was performed based on the expected number of haplotypes on each individual. A test statistic was computed from genotype that generates sets of haplotype-specific tests with 1 degree of freedom. P-values were generated for each haplotype to observe the significant changes in haplotype distribution for both cases and unrelated controls^18^.

### Identity by descent (IBD) analysis

IBD analysis was performed to identify relatedness between SG probands and family cohort. IBD was estimated on sequence data using—genome option in PLINK^15^. Plink –genome calculates identity by state (IBS) for each pair of individuals based on the average proportions of alleles shared at genotyped SNPs and estimates pairwise kinship. The degree of recent shared ancestry (IBD) is estimated from the genome-wide IBS. This approach determines the proportion of loci shares between two individuals to be zero alleles (Z0), one allele (Z1), or two alleles (Z = 2). The proportion of IBD = P(IBD = 2) + 0.5*P(IBD = 1) between the two individuals is returned as PI-HAT. Consecutively, using R (version 4.2.2)^19^, Z0 against PI-HAT were plotted to estimate relatedness between the two individual pairs.

### Estimation of inbreeding coefficient

Inbreeding co-efficient (F-value) of SNP markers from chromosome 4 region (risk haplotype region) were separately estimated for the founders of SG cohort (732 markers), unrelated controls of north Indian population (932 markers) and south Indian population (853 markers) using PLINK^15^. Inbreeding coefficient (F) value was calculated using formula (F = expected heterozygosity − observed heterozygosity/expected heterozygosity). The heterozygosity of the SNP markers was estimated based on the F-value calculated. Furthermore, using R (version 4.2.2)^19^, F-value distribution was plotted to measure the proportion of heterozygosity in all three cohorts and compare the inbreeding among them.

### Ethics approval

The study was approved by the National Institute of Mental Health and Neurosciences, Bangalore ethics committee (NIMHANS/IEC/2020-21). Written informed consent was obtained from the parents and/or legal guardian of all participants in the study.

## Results

### Haplotype reconstruction

**Figure 1:**
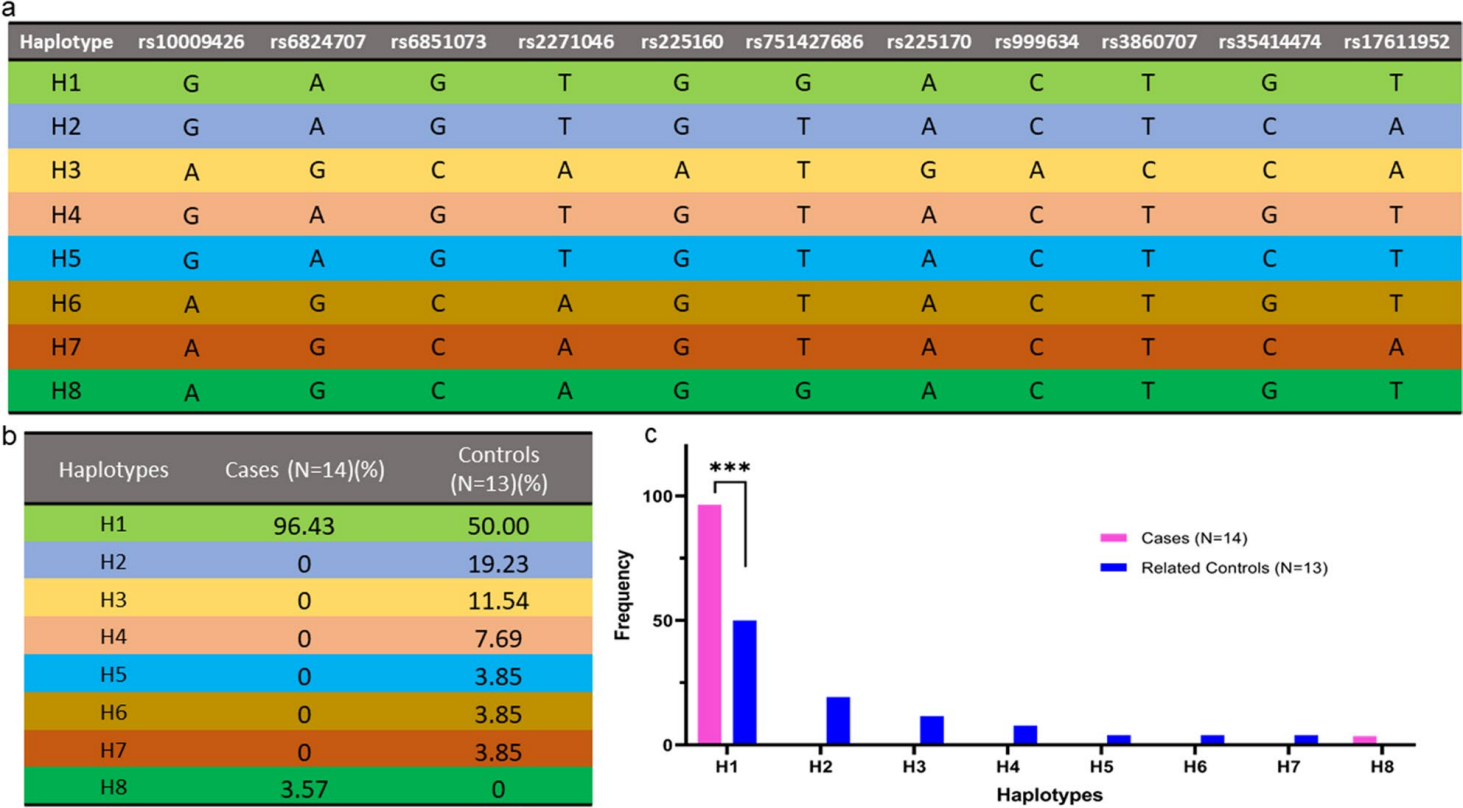
(**a**) The eight haplotypes reconstructed from the SNPs rs10009426, rs6824707, rs6851073, rs2271046, rs225160, rs225170, rs999634, rs3860707, rs35414474 and rs17611952 selected as neutral markers surrounding c.544 T > G mutation. Each haplotype is colour coded. (**b**) The frequency of the haplotypes in cases and unaffected related controls. (**c**) Graphical distribution of haplotype percentages in cases and unaffected related controls.

Haplotype reconstruction based on the 10 markers selected including the c. 544 T > G mutation (rs10009426, rs6824707, rs6851073, rs2271046, rs225160, rs225170, rs999634, rs3860707, rs35414474 and rs17611952) for the two groups (cases and related controls) were conducted. The genotypes of 10 markers selected surrounding the c.544 T > G (rs751427686) mutation for the cases and unaffected related controls are described in Table S1. A total of 8 haplotypes were estimated in cases and related controls named as H1 (G, A, G, T, G, G, A, C, T, G, T), H2 (G, A, G, T, G T, A, C, T, C, A), H3 (A, G, C, A, A, T, G, A, C, C, A), H4 (G, A, G, T, G, T, A, C, T, G, T), H5 (G, A, G, T, G, T, A, C, T, C, T), H6 (A, G, C, A, G, T, A, C, T, G, T) H7 (A, G, C, A, G, T, A, C, T, C, A) and H8 (A, G, C, A, G, G, A, C, T, G, T) (Fig. 1a). In the cases, only H1 and H8 haplotype were identified with frequency of 96.43% and 3.57% respectively. In the related controls, the frequency of H1 was 50% while H8 was absent (Fig. 1b,c).

**Figure 2:**
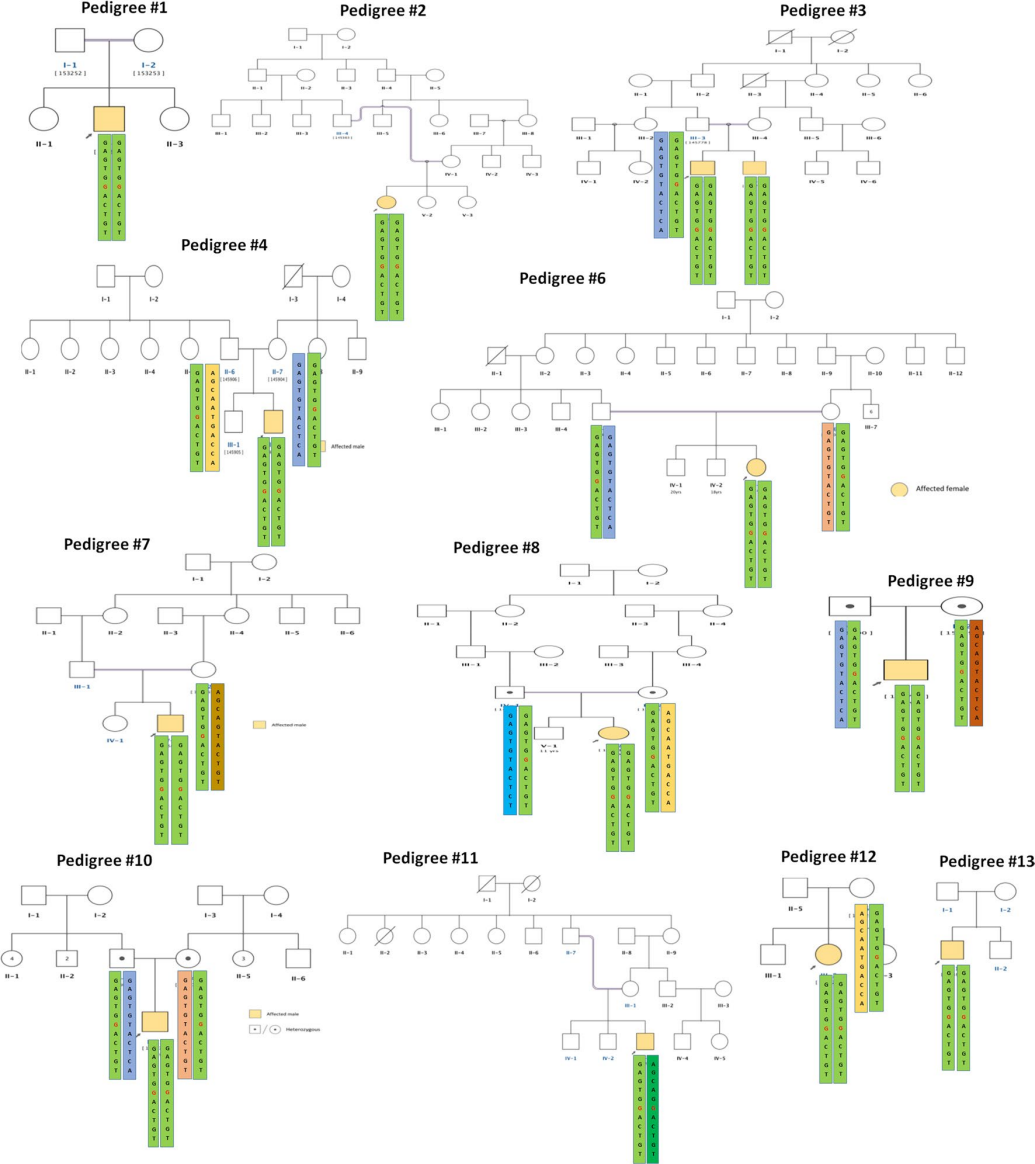
The pedigrees of the 14 cases (including a pair of siblings) showing the distribution of the eight haplotypes identified.

The H1 haplotype was present in homozygous state in all the cases including a pair of affected siblings in pedigree 3 carrying c. 544 T > G mutation except patient 11, indicating a strong association of this haplotype with the disease. In patient 11, H1 is found to occur heterozygously with H8 which differs in the alleles at rs10009426, rs6824707, rs6851073 and rs2271046 indicating a possible single recombination event between rs2271046 and rs225160 in patient SG-11. In the related controls, H1 is found to occur in heterozygous state along with other haplotypes (Fig. 2). The frequency of the other haplotypes identified in related controls were H2 (19.23%), H3 (11.54%), H4 (7.69%), H5 (3.85%), H6 (3.85%) and H7 (3.85%) (Fig. 1b,c).

**Figure 3:**
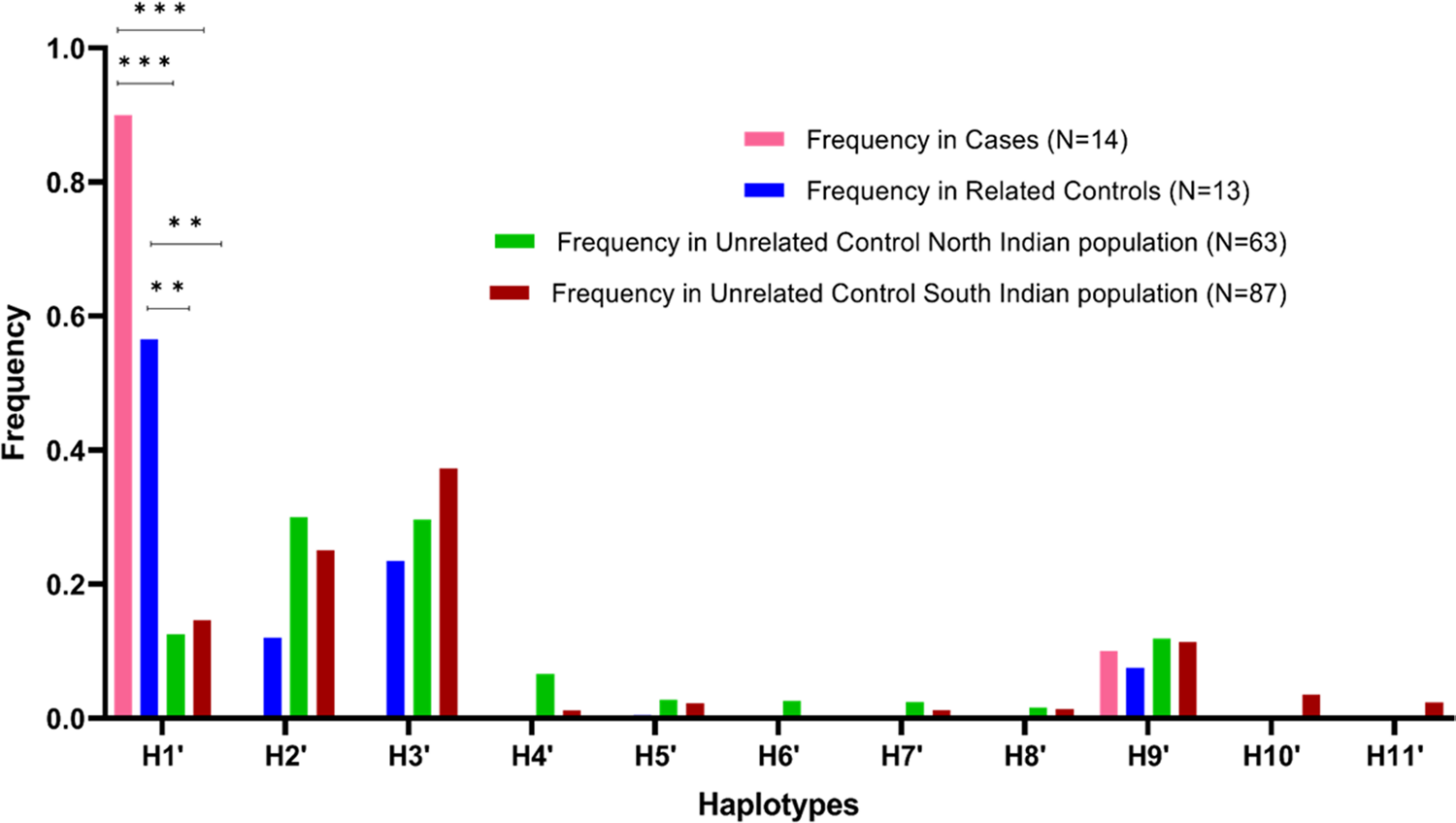
Graphical distribution of haplotype frequencies in cases, related controls and unrelated control north and south Indian populations.

To assess if H1 haplotype is strongly associated with the disease and is indeed the result of founder effect, genotypes of 87 unrelated controls from south and 63 unrelated controls from north Indian populations were taken from the GenomeAsia 100 K^16^ (Table S2). A new set of haplotypes were constructed in the background of c. 544 T > G including the same SNPs rs10009426, rs6824707, rs6851073, rs2271046, rs225160, rs225170, rs999634, rs3860707, rs35414474 and rs17611952. Their frequencies were estimated in the cases, related controls and the 150 unrelated controls from south and north Indian populations (Table S3). Eleven haplotypes were identified: H1’ (G, A, G, T,G, A, C, T, G, T), H2’ (A, G,C,,A, A, G, A, C, C, A), H3’ (G, A, G, T, G, A,C, T, C, A), H4’ (G, A, G, T,G, A, C, C, C, A), H5’ (A, G,C, A, G, A, C, T,C, A), H6’ (A, G, C, A, A, G, A, T, C, A), H7’ (G, A, G, T, G, A, A, C, C, A), H8’ (A, G, C, A, A, G, C, T, C, A), H9’ (A, G, C, A, G, A, C, T, G, T), H10’ (A, G, C, A, G, A, C, C, C, A) H11’ (A, G, C, A, A, G, C, C, C, A) (Table 3). The H1’ haplotype was found to occur significantly in higher frequency in the cases compared to the unrelated control south Indian population (p-value 3.00E^−09^) (Fig. 3) and unrelated control north Indian population (p-value 1.12E^−09^) (Fig. 3). Similarly, the H1’ haplotype was also observed to occur in frequency significantly higher in related controls compared to the unrelated control south Indian population (p-value 1.34E^−06^) and unrelated control north Indian population (p-value 5.17E^−07^) (Fig. 3). The significant higher expression of H1’ haplotype in the patients compared to unrelated control north and south Indian populations indicates this haplotype as an ancestral haplotype shared by the patients.

**Table 3:** The eleven haplotypes identified in SG cases, related controls and unrelated general south and north Indian population.

Haplotype	rs10009426	rs6824707	rs6851073	rs2271046	rs225160	rs225170	rs999634	rs3860707	rs35414474	rs17611952
H1’	G	A	G	T	G	A	C	T	G	T
H2’	A	G	C	A	A	G	A	C	C	A
H3’	G	A	G	T	G	A	C	T	C	A
H4’	G	A	G	T	G	A	C	C	C	A
H5’	A	G	C	A	G	A	C	T	C	A
H6’	A	G	C	A	A	G	A	T	C	A
H7’	G	A	G	T	G	A	A	C	C	A
H8’	A	G	C	A	A	G	C	T	C	A
H9’	A	G	C	A	G	A	C	T	G	T
H10’	A	G	C	A	G	A	C	C	C	A
H11’	A	G	C	A	A	G	C	C	C	A

### IBD analyses between SG families

**Figure 4:**
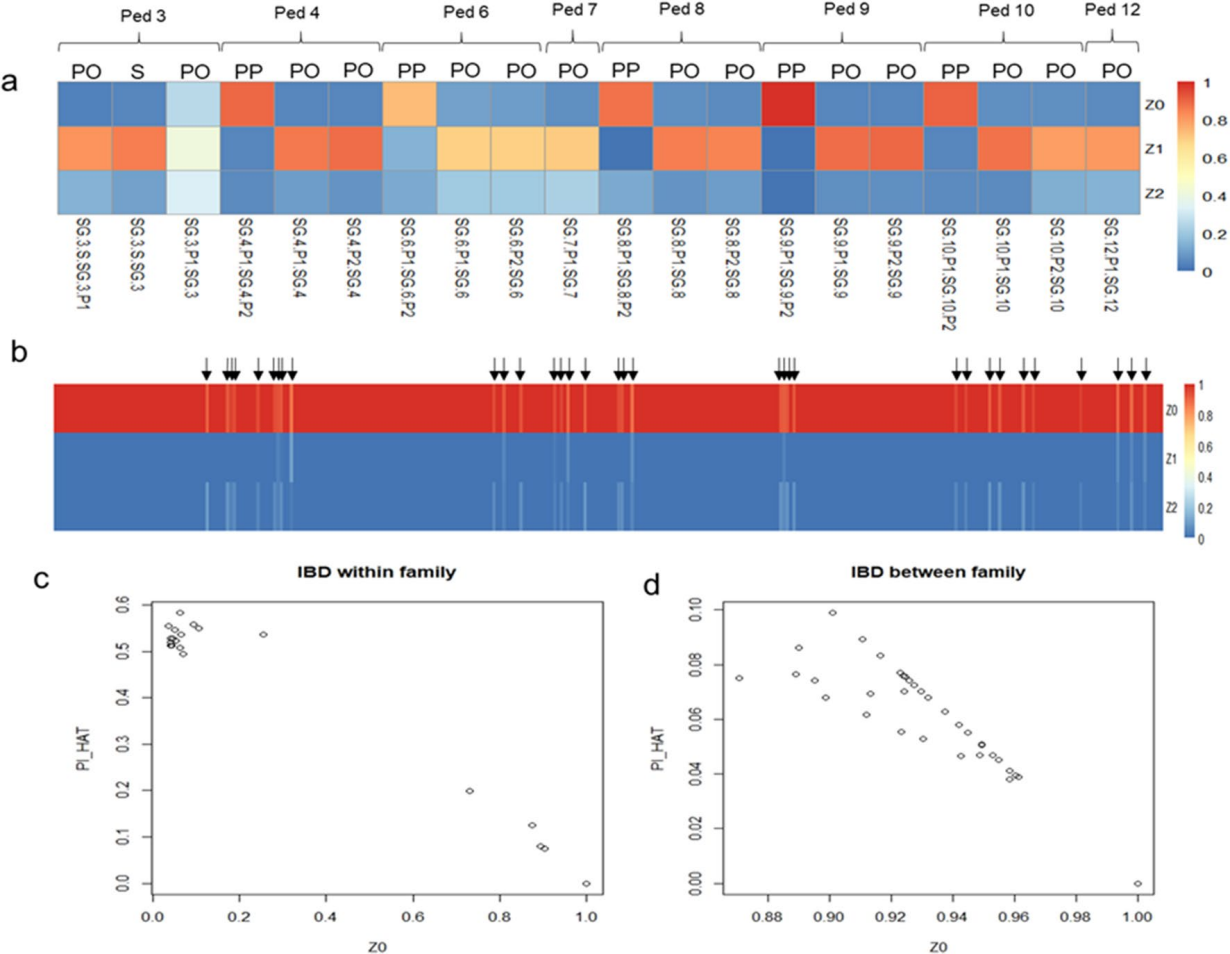
Proportion of loci shares to be zero alleles (Z0), one allele (Z1), or two alleles (Z = 2) (**a**) between individual pairs of the same family pedigree (**b**) between individual pairs of different family pedigrees. Arrows indicate the seemingly unrelated individual pairs sharing a novel relationship with Z1 and Z2 IBD share; (**c**) Z0 against PI-HAT plot to estimate relatedness between two individual pairs within same family pedigree (**d**) different family pedigree. *PO* Parent-offspring, *S* Siblings, *PP* Parent1–Parent2.

The 27 individuals in our SG cohort included 14 cases and 13 related family members from 13 different families. The 14 parent–offspring pairs from pedigrees 3, 4, 6, 7, 8, 9, 10 and 12 shared higher proportion of loci with one allele share (mean Z1 = 0.78), two allele share (mean Z2 = 0.13), lower proportion of loci with zero allele share (mean Z0 = 0.07) with higher PI-HAT (mean PI-HAT = 0.53) values. The IBD analysis also revealed degree of relatedness between 4 pairs of parents from pedigrees 4, 6, 8 and 10 with proportion of one allele share (mean Z1 = 0.05) and two alleles shares (mean Z2 = 0.09) with moderate PI-HAT values (mean PI-HAT = 0.12). Pedigree 9 was the only family of non-consanguineous parentage sharing no IBD between parents (Z0 = 1, Z1 = 0, Z2 = 0) (Fig. 4a,c) (Table S4).

The IBD analysis was extended to identify relationships between seemingly unrelated individuals between the 13 families in our cohort. A total of all possible 329 pairs of unrelated individuals were analysed to identify any shared proportion of IBD between them. Of these, 296 pairs did not share any IBD (Z0 = 1, Z1 = 0, Z2 = 0). However, 33 pairs of seemingly unrelated individuals were identified that shared IBD (mean Z1 = 0.01, mean Z2 = 0.05, mean PI-HAT = 0.06) (Table 4) (Table S5). These individuals belonged to the pedigrees 2, 3, 4, 6, 8, 9, 10 and 12. The Z0 and PI-HAT for these 33 individual pairs are plotted in Fig. 4b,d.

**Table 4:** Identification of related pairs between families in the SG cohort.

S.N	FID1	IID1	FID2	IID2
1	Ped2	SG-2	Ped10	SG-10-P2
2	Ped3	SG-3-S	Ped4	SG-4-P1
3	Ped3	SG-3-S	Ped4	SG-4-P2
4	Ped3	SG-3-S	Ped4	SG-4
5	Ped3	SG-3-S	Ped8	SG-8-P1
6	Ped3	SG-3-S	Ped9	SG-9
7	Ped3	SG-3-S	Ped10	SG-10-P1
8	Ped3	SG-3-S	Ped10	SG-10-P2
9	Ped3	SG-3-S	Ped12	SG-12-P1
10	Ped4	SG-4-P1	Ped10	SG-10-P2
11	Ped4	SG-4-P1	Ped12	SG-12-P1
12	Ped4	SG-4-P2	Ped6	SG-6-P2
13	Ped4	SG-4-P2	Ped10	SG-10-P1
14	Ped4	SG-4-P2	Ped10	SG-10
15	Ped4	SG-4-P2	Ped12	SG-12-P1
16	Ped4	SG-4	Ped6	SG-6-P2
17	Ped4	SG-4	Ped10	SG-10-P1
18	Ped4	SG-4	Ped10	SG-10-P2
19	Ped4	SG-4	Ped12	SG-12-P1
20	Ped6	SG-6-P2	Ped10	SG-10-P1
21	Ped6	SG-6-P2	Ped10	SG-10-P2
22	Ped6	SG-6-P2	Ped10	SG-10
23	Ped6	SG-6-P2	Ped12	SG-12-P1
24	Ped8	SG-8-P1	Ped10	SG-10-P2
25	Ped8	SG-8-P1	Ped12	SG-12-P1
26	Ped8	SG-8-P2	Ped10	SG-10-P2
27	Ped8	SG-8-P2	Ped12	SG-12-P1
28	Ped8	SG-8	Ped10	SG-10-P2
29	Ped8	SG-8	Ped12	SG-12-P1
30	Ped9	SG-9-P2	Ped12	SG-12-P1
31	Ped10	SG-10-P1	Ped12	SG-12-P1
32	Ped10	SG-10-P2	Ped12	SG-12-P1
33	Ped10	SG-10	Ped12	SG-12-P1

*FID1* Family ID 1, *FID2* Family ID 2, *IID1* Individual ID 1, *IID2* Individual ID 2.

### Determination of inbreeding coefficient in the SG cohort

**Figure 5:**
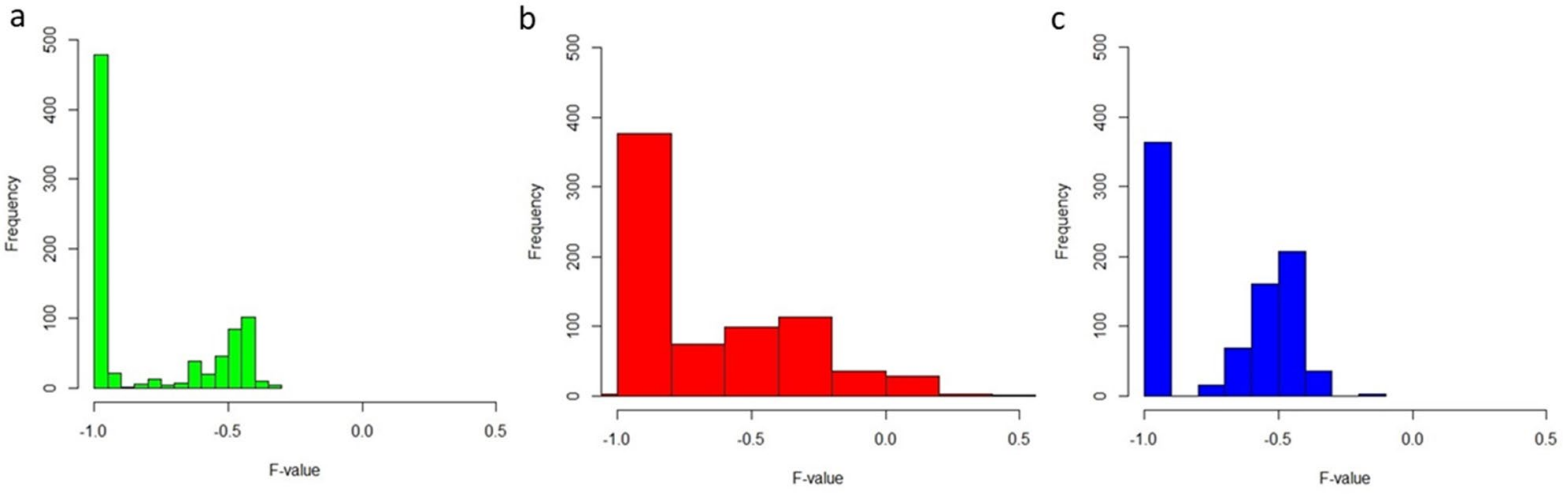
Distribution of F-values (**a**) North Indian population (932 markers); (**b**) Founders of SG cohort (732 markers) and (**c**) South Indian population (853 markers).

In the founder population of SG cohort, distribution of F value as inbreeding coefficient ranges from − 1.1 to 0.28. In the general north and south Indian population, the distribution of F-value ranges from − 1 to − 0.3 and − 1 to − 0.2, respectively, indicating heterozygotes superiority. The distribution of the F-values is plotted for all the three groups separately (Fig. 5).

## Discussions

In this study, we describe a shared, common haplotype co-segregating in 14 genetically and clinically confirmed SG cases reported by Bardhan et al. from 13 unrelated families from south Indian region with the likely pathogenic homozygous mutation c.544 T > G in *SGCB* gene^8^. This mutation has also been previously reported by Ganapathy et al.^20^ in two patients one in homozygous state and one in compound heterozygous state with another mutation (c.286G > C) in the *SGCB* gene. This extremely rare variant (c.544 T > G, p.Thr182Pro) has been reported in dbSNP (rs751427686), and totally absent in the 1000 genome phase 3 data. However, in the GnomAD database, this rare variant is present with extremely low frequency (1.193e^−05^ in all and 9.8e^−05^ in SAS; Table 2). The 182nd Threonine residue is located in the extracellular domain of the beta component of Sarcoglycan protein. Changing this to Proline due to the c.544 T > G mutation is therefore creating a deleterious and potentially damaging change in the protein, thus making it a likely pathogenic genetic alteration. Furthermore, to the best of our knowledge, this mutation, in the context of sarcoglycanopathies, is not reported in any other population, thereby ruling out the hypothesis that it is caused by a mutation ‘hotspot’. Presence of this mutation in homozygous condition in 14 patients from 13 unrelated families therefore, naturally qualify this as a founder mutation and we wanted to probe further whether the patients harbouring this mutation also share a common founder haplotype at the *SGCB* locus.

We identified the H1 (G, A, G, T, G, G, A, C, T, G, T) haplotype formed by 10 markers including the c.544 T > G mutation (rs10009426, rs6824707, rs6851073, rs2271046, rs225160, rs751427686, rs225170, rs999634, rs3860707, rs35414474 and rs17611952) to segregate in a homozygous state in all the cases except patient 11 (Figs. 1, 2). This indicates a strong association of this haplotype with the disease. We also identified haplotype H1 to occur with haplotype H8 (A, G, C, A, G, G, A, C, T, G, T) in patient 11 indicating a possible single recombination event between rs2271046 and rs225160. The H1 haplotype is present in all the unaffected carriers in heterozygous condition in combination with other haplotypes (H2, H3, H4, H5, H6 and H7) (Fig. 2). The homozygous occurrence of the H1 haplotype in SG cases with clear indication of its segregation pattern in their families designate this as the primary risk haplotype co-segregating with the c.544 T > G mutation in the *SGCB* gene.

Additionally, to assess if this risk haplotype is indeed the result of a founder event, we again reconstructed haplotypes with the same set of SNP markers in the background of the mutation c.544 T > G in 150 unrelated control samples (87 south and 63 north Indian population). The genotypes of these unrelated control populations were taken from the GenomeAsia 100 K^16^. The H1’ haplotype which is similar to the earlier H1 haplotype, except the mutation c.544 T > G, has also been found to be in significantly higher frequency in cases and related control group compared to the unrelated control Indian population (Fig. 3). The higher frequency of this risk haplotype in the cases and related controls indicate a clear recent founder effect. The study would probably have a better accuracy with a higher sample size for c.544 T > G mutation carriers, but we were limited in part by the fact that sarcoglycanopathy is a rare disease.

In literature, several reports have identified founder effect of frequently occurring mutations in SG. For example, in 8 families from the south-east region of Iran and the Baloch ethnic group, a homozygous mutation leading to the deletion of exon 2 in *SGCB* gene was identified. Haplotype analysis were based on 4 STR markers surrounding the deletion and was found to be homozygous in all the patients^21^. In another study by Alavi et al. 12 SG patients from south and south-east Iran had a deletion encompassing whole of exon 2 in *SGCB* gene. Haplotype analysis was done based on 3 SNPs surrounding the deletion. The genotypes of the markers were found to be homozygous in all the patients indicating founder effect^22^.

To the best of our knowledge, ours is the first such study reporting founder effect of mutation c.544 T > G in *SGCB* gene in Indian population. To corroborate our findings, we additionally performed an IBD analysis which is a powerful method that identifies relatedness between individual pairs by inferring regions of the genome that have been inherited from common ancestors^10^. Identifying clusters of individuals sharing common allele through IBD therefore indicates common ancestry. We hypothesized that there might be unknown relatedness in our cohort between individuals of SG families. IBD analysis was performed on sequencing data of the SG cases and related controls within the families as well as between the families. The relatedness within the family members of the pedigree was known as indicated in Table 1. IBD analysis correctly confirmed the degree of relatedness between the parent-offspring, siblings as well as between parents (Fig. 4a). The 14 known parents-offspring relationships shared a lower Z0 value, higher Z1 value and a higher PI-HAT value as expected (Fig. 4a,c). To measure the degree of relatedness between parents of the families, 5 pairs of parents from pedigrees 4, 6, 8, 9 and 10 were analysed. Pedigrees 6 and 8 were consanguineous families while the rest were non-consanguineous families. Except for pedigree 9 in which the parents shared no IBD (Z0 = 1, Z1 = 0, Z2 = 0), the parents in the other four pedigrees 4, 6, 8 and 10 shared IBD thereby confirming relatedness between them (Fig. 4a). Additionally, of the 329 seemingly unrelated individual pairs between families, IBD analysis revealed sharing of one allele (Z1) as well as 2 alleles (Z2) throughout the genome of 33 pairs of individuals thereby indicating relatedness between them (Fig. 4b,d). These pairs belonged to the pedigrees 2, 3, 4, 6, 8, 9, 10 and 12 as indicated in Table 1. The other 288 pairs did not share any IBD with Z0 = 1. Identification of relatedness in these 33 pairs of individual’s cluster in our SG cohort support the origin of the risk haplotype H1 from a common ancestral founder.

We also performed inbreeding coefficient analysis that measures the fraction by which the heterozygosity of markers has been reduced due to inbreeding leading to the increase of homozygosity^23^. A substantial proportion of the marker inbreeding coefficient estimates are expected to be negative. However, F estimates can become biased when the sample contain high proportion of inbred and/or closely related individuals^24^. We estimated the inbreeding coefficient of SNP markers from chromosome 4 region for the founders of SG cohort as well as the unrelated controls of north and south Indian populations. We observed a significant proportion of marker estimates higher than zero in the founders of SG cohort, indicating homozygosity superiority, although in the unrelated control north and south Indian population, the marker estimates are negative, indicating heterozygosity superiority.

Our results of IBD analysis and estimation of inbreeding coefficient analysis corroborate the high rate of consanguinity that has resulted in over-representation of this mutation in our cohort (71.4% of the patients are from families with consanguineous parentage). Since all these patients are from the south Indian region of Tamil Nadu, Karnataka and Andhra Pradesh, we suggest this region to be a primary target of mutation screening in patients diagnosed with SG. Consecutively, larger cohort with multiple patients from different geographical regions and ethnicities need to be screened to achieve reliable and comprehensive data for genetically informed risk stratification and better management of disease.

### Supplementary Information

Supplementary Information.

## Data Availability

The data that support the findings of this study are available from the corresponding author at doi: https://doi.org/10.1007/s10048-022-00690-9^25^ and GenomeAsia 100 K dataset at https://doi.org/10.1038/s41586-019-1793-z^16^.
