# Supplementary material for: Identification of a shared, common haplotype segregating with an SGCB c.544 T > G mutation in Indian patients affected with sarcoglycanopathy
Source: Sci Rep. 2023 Sep 12;13:15095. doi: 10.1038/s41598-023-41487-6 (PMC10497502; doi:10.1038/s41598-023-41487-6)
Supplement: Supplementary file 1 — Supplementary Information. [file 41598_2023_41487_MOESM1_ESM.docx]

**Supplementary Information**

**S1 figure: Comprehensive clinical details of sarcoglycanopathy cases in our cohort.**

**
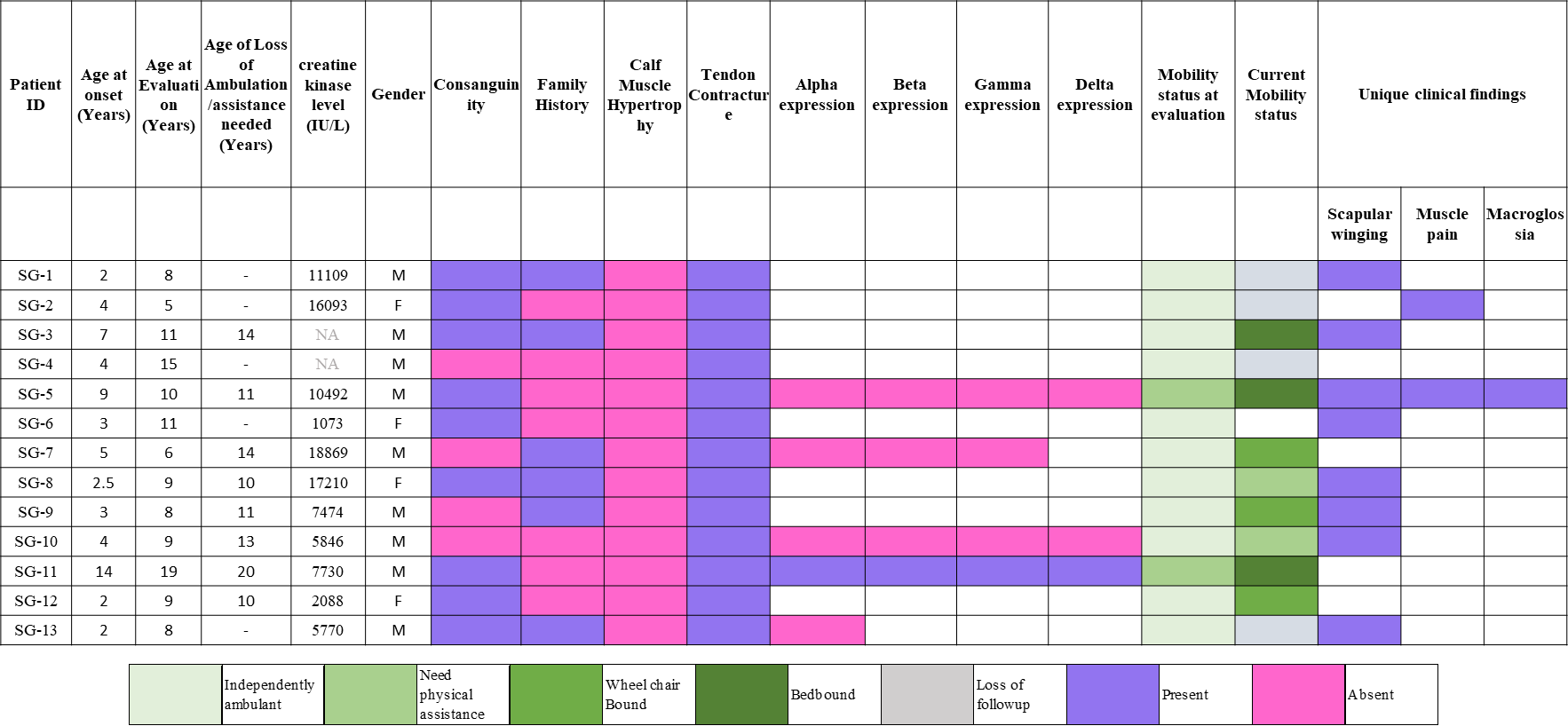
**

**S1 table:** The genotypes of 10 markers selected surrounding the c.544T>G (p.Thr182Pro) (rs751427686) mutation for the cases and unaffected related controls.

| **ID** | **rs10009426** | **rs6824707** | **rs6851073** | **rs2271046** | **rs225160** | **rs751427686** | **rs225170** | **rs999634** | **rs3860707** | **rs35414474** | **rs17611952** |
| --- | --- | --- | --- | --- | --- | --- | --- | --- | --- | --- | --- |
| SG-1 | G/G | A/A | G/G | T/T | G/G | G/G | A/A | C/C | T/T | G/G | T/T |
| SG-2 | G/G | A/A | G/G | T/T | G/G | G/G | A/A | C/C | T/T | G/G | T/T |
| SG-3 | G/G | A/A | G/G | T/T | G/G | G/G | A/A | C/C | T/T | G/G | T/T |
| SG-3-S | G/G | A/A | G/G | T/T | G/G | G/G | A/A | C/C | T/T | G/G | T/T |
| SG-3-P1 | G/G | A/A | G/G | T/T | G/G | T/G | A/A | C/C | T/T | C/G | A/T |
| SG-4 | G/G | A/A | G/G | T/T | G/G | G/G | A/A | C/C | T/T | G/G | T/T |
| SG-4-P1 | A/G | G/A | C/G | A/T | G/A | T/G | A/G | C/A | T/C | C/G | A/T |
| SG-4-P2 | G/G | A/A | G/G | T/T | G/G | T/G | A/A | C/C | T/T | C/G | A/T |
| SG-5 | G/G | A/A | G/G | T/T | G/G | G/G | A/A | C/C | T/T | G/G | T/T |
| SG-6 | G/G | A/A | G/G | T/T | G/G | G/G | A/A | C/C | T/T | G/G | T/T |
| SG-6-P1 | G/G | A/A | G/G | T/T | G/G | T/G | A/A | C/C | T/T | G/G | T/T |
| SG-6-P2 | G/G | A/A | G/G | T/T | G/G | T/G | A/A | C/C | T/T | C/G | A/T |
| SG-7 | G/G | A/A | G/G | T/T | G/G | G/G | A/A | C/C | T/T | G/G | T/T |
| SG-7-P1 | A/G | G/A | C/G | A/T | G/G | T/G | A/A | C/C | T/T | G/G | T/T |
| SG-8 | G/G | A/A | G/G | T/T | G/G | G/G | A/A | C/C | T/T | G/G | T/T |
| SG-8-P1 | A/G | G/A | C/G | A/T | G/A | T/G | A/G | C/A | T/C | C/G | A/T |
| SG-8-P2 | G/G | A/A | G/G | T/T | G/G | T/G | A/A | C/C | T/T | C/G | T/T |
| SG-9 | G/G | A/A | G/G | T/T | G/G | G/G | A/A | C/C | T/T | G/G | T/T |
| SG-9-P1 | A/G | G/A | C/G | A/T | G/G | T/G | A/A | C/C | T/T | C/G | A/T |
| SG-9-P2 | G/G | A/A | G/G | T/T | G/G | T/G | A/A | C/C | T/T | C/G | A/T |
| SG-10 | G/G | A/A | G/G | T/T | G/G | G/G | A/A | C/C | T/T | G/G | T/T |
| SG-10-P1 | G/G | A/A | G/G | T/T | G/G | T/G | A/A | C/C | T/T | C/G | A/T |
| SG-10-P2 | G/G | A/A | G/G | T/T | G/G | T/G | A/A | C/C | T/T | G/G | T/T |
| SG-11 | A/G | G/A | C/G | A/T | G/G | G/G | A/A | C/C | T/T | G/G | T/T |
| SG-12 | G/G | A/A | G/G | T/T | G/G | G/G | A/A | C/C | T/T | G/G | T/T |
| SG-12-P1 | A/G | G/A | C/G | A/T | G/A | T/G | A/G | C/A | T/C | C/G | A/T |
| SG-13 | G/G | A/A | G/G | T/T | G/G | G/G | A/A | C/C | T/T | G/G | T/T |

**S2 table:** The genotypes of 10 markers selected surrounding the c.544T>G (p.Thr182Pro) (rs751427686) mutation for the south Indian population (N=87) and north Indian population (N=63).

South Indian population:

|  | **rs10009426** | **rs6824707** | **rs6851073** | **rs2271046** | **rs225160** | **rs225170** | **rs999634** | **rs3860707** | **rs35414474** | **rs17611952** |
| --- | --- | --- | --- | --- | --- | --- | --- | --- | --- | --- |
| 1 | A/G | G/A | C/G | A/T | A/G | G/A | C/C | T/T | G/G | T/T |
| 2 | A/A | G/G | C/C | A/A | G/G | A/A | C/C | T/T | G/G | T/T |
| 3 | G/G | A/A | G/G | T/T | G/G | A/A | C/C | T/T | C/C | A/A |
| 4 | A/A | G/G | C/C | A/A | A/G | G/A | A/C | C/T | G/C | T/A |
| 5 | G/G | A/A | G/G | T/T | G/G | A/A | C/C | T/T | C/C | A/A |
| 6 | A/A | G/G | C/C | A/A | A/G | G/A | C/C | T/T | G/C | T/A |
| 7 | A/A | G/G | C/C | A/A | A/A | G/G | A/C | C/C | C/C | A/A |
| 8 | A/G | G/A | C/G | A/T | A/G | G/A | A/C | C/T | C/C | A/A |
| 9 | A/G | G/A | C/G | A/T | A/G | G/A | C/C | T/T | C/C | A/A |
| 10 | G/G | A/A | G/G | T/T | G/G | A/A | C/C | T/T | G/C | T/A |
| 11 | G/G | A/A | G/G | T/T | G/G | A/A | C/C | C/T | G/C | T/A |
| 12 | A/A | G/G | C/C | A/A | A/G | G/A | A/C | C/T | G/C | T/A |
| 13 | A/G | G/A | C/G | A/T | G/G | A/A | C/C | T/T | G/C | T/A |
| 14 | A/G | G/A | C/G | A/T | G/G | A/A | C/C | T/T | G/C | T/A |
| 15 | A/A | G/G | C/C | A/A | A/G | G/A | A/C | C/T | G/C | T/A |
| 16 | A/G | G/A | C/G | A/T | A/G | G/A | A/C | C/C | C/C | A/A |
| 17 | A/G | G/A | C/G | A/T | A/G | G/A | A/C | C/T | C/C | A/A |
| 18 | A/A | G/G | C/C | A/A | A/A | G/G | A/A | C/C | C/C | A/A |
| 19 | A/A | G/G | C/C | A/A | A/G | G/A | A/C | T/T | C/C | A/A |
| 20 | G/G | A/A | G/G | T/T | G/G | A/A | C/C | T/T | G/C | T/A |
| 21 | A/G | G/A | C/G | A/T | A/G | G/A | C/C | T/T | G/C | T/A |
| 22 | A/G | G/A | C/G | A/T | G/G | A/A | C/C | T/T | G/C | T/A |
| 23 | G/G | A/A | G/G | T/T | G/G | A/A | C/C | T/T | C/C | A/A |
| 24 | A/A | G/G | C/C | A/A | A/A | G/G | A/A | C/C | C/C | A/A |
| 25 | A/G | G/A | C/G | A/T | A/G | G/A | A/C | C/T | G/C | T/A |
| 26 | G/G | A/A | G/G | T/T | G/G | A/A | C/C | T/T | C/C | A/A |
| 27 | A/G | G/A | C/G | A/T | A/G | G/A | A/C | C/T | C/C | A/A |
| 28 | G/G | A/A | G/G | T/T | G/G | A/A | C/C | T/T | G/C | T/A |
| 29 | G/G | A/A | G/G | T/T | G/G | A/A | C/C | T/T | C/C | A/A |
| 30 | A/G | G/A | C/G | A/T | G/G | A/A | C/C | T/T | C/C | A/A |
| 31 | A/G | G/A | C/G | A/T | A/G | G/A | A/C | C/T | C/C | A/A |
| 32 | A/G | G/A | C/G | A/T | A/G | G/A | A/A | C/C | C/C | A/A |
| 33 | G/G | A/A | G/G | T/T | G/G | A/A | C/C | T/T | C/C | A/A |
| 34 | G/G | A/A | G/G | T/T | G/G | A/A | C/C | T/T | G/G | T/T |
| 35 | G/G | A/A | G/G | T/T | G/G | A/A | C/C | T/T | C/C | A/A |
| 36 | A/G | G/A | C/G | A/T | A/G | G/A | A/C | C/T | G/C | T/A |
| 37 | G/G | A/A | G/G | T/T | G/G | A/A | C/C | T/T | C/C | A/A |
| 38 | G/G | A/A | G/G | T/T | G/G | A/A | C/C | T/T | G/C | T/A |
| 39 | G/G | A/A | G/G | T/T | G/G | A/A | C/C | T/T | G/C | T/A |
| 40 | G/G | A/A | G/G | T/T | G/G | A/A | C/C | T/T | G/G | T/T |
| 41 | A/G | G/A | C/G | A/T | A/G | G/A | A/C | C/T | C/C | A/A |
| 42 | A/G | G/A | C/G | A/T | G/G | A/A | C/C | T/T | G/G | T/T |
| 43 | A/G | G/A | C/G | A/T | A/G | G/A | A/C | C/T | C/C | A/A |
| 44 | A/G | G/A | C/G | A/T | A/G | G/A | A/C | C/T | G/C | T/A |
| 45 | A/A | G/G | C/C | A/A | A/G | G/A | A/C | C/T | G/C | T/A |
| 46 | A/G | G/A | C/G | A/T | A/G | G/A | A/C | C/T | C/C | A/A |
| 47 | A/G | G/A | C/G | A/T | A/G | G/A | A/C | C/T | C/C | A/A |
| 48 | A/A | G/G | C/C | A/A | A/A | G/G | A/A | C/C | C/C | A/A |
| 49 | A/A | G/G | C/C | A/A | A/A | G/G | A/A | C/C | C/C | A/A |
| 50 | G/G | A/A | G/G | T/T | G/G | A/A | C/C | T/T | C/C | A/A |
| 51 | G/G | A/A | G/G | T/T | G/G | A/A | C/C | T/T | C/C | A/A |
| 52 | A/G | G/A | C/G | A/T | A/G | G/A | A/C | C/T | C/C | A/A |
| 53 | A/G | G/A | C/G | A/T | A/G | G/A | A/C | C/T | C/C | A/A |
| 54 | G/G | A/A | G/G | T/T | G/G | A/A | C/C | T/T | C/C | A/A |
| 55 | A/G | G/A | C/G | A/T | A/G | G/A | A/C | C/T | C/C | A/A |
| 56 | G/G | A/A | G/G | T/T | G/G | A/A | C/C | T/T | G/C | T/A |
| 57 | G/G | A/A | G/G | T/T | G/G | A/A | C/C | T/T | G/G | T/T |
| 58 | A/G | G/A | C/G | A/T | A/G | G/A | A/C | C/T | C/C | A/A |
| 59 | G/G | A/A | G/G | T/T | G/G | A/A | C/C | T/T | C/C | A/A |
| 60 | A/G | G/A | C/G | A/T | A/G | G/A | A/C | C/T | G/C | T/A |
| 61 | A/A | G/G | C/C | A/A | G/G | A/A | C/C | C/T | G/C | T/A |
| 62 | A/G | G/A | C/G | A/T | A/G | G/A | A/C | C/T | C/C | A/A |
| 63 | A/G | G/A | C/G | A/T | G/G | A/A | C/C | T/T | G/C | T/A |
| 64 | A/A | G/G | C/C | A/A | A/G | G/A | A/C | C/C | C/C | A/A |
| 65 | A/G | G/A | C/G | A/T | G/G | A/A | C/C | T/T | G/C | T/A |
| 66 | A/A | G/G | C/C | A/A | A/G | G/A | C/C | C/C | C/C | A/A |
| 67 | A/G | G/A | C/G | A/T | G/G | A/A | C/C | C/T | C/C | A/A |
| 68 | A/A | G/G | C/C | A/A | A/G | G/A | A/C | C/C | C/C | A/A |
| 69 | A/G | G/A | C/G | A/T | A/G | G/A | A/C | C/T | G/C | T/A |
| 70 | A/G | G/A | C/G | A/T | G/G | A/A | C/C | C/T | C/C | A/A |
| 71 | A/G | G/A | C/G | A/T | A/G | G/A | A/C | C/T | G/C | T/A |
| 72 | A/A | G/G | C/C | A/A | A/A | G/G | A/C | C/C | C/C | A/A |
| 73 | A/A | G/G | C/C | A/A | G/G | A/A | C/C | T/T | G/G | T/T |
| 74 | A/G | G/A | C/G | A/T | G/G | A/A | C/C | T/T | G/C | T/A |
| 75 | A/A | G/G | C/C | A/A | A/G | G/A | A/C | C/T | G/C | T/A |
| 76 | A/G | G/A | C/G | A/T | A/G | G/A | C/C | C/T | C/C | A/A |
| 77 | G/G | A/A | G/G | T/T | G/G | A/A | C/C | T/T | C/C | A/A |
| 78 | A/G | G/A | C/G | A/T | A/G | G/A | A/C | C/T | C/C | A/A |
| 79 | G/G | A/A | G/G | T/T | G/G | A/A | C/C | T/T | G/C | T/A |
| 80 | A/G | G/A | C/G | A/T | A/G | G/A | A/C | C/T | C/C | A/A |
| 81 | A/G | G/A | C/G | A/T | A/G | G/A | A/C | C/T | C/C | A/A |
| 82 | A/G | G/A | C/G | A/T | G/G | A/A | A/C | C/T | G/C | T/A |
| 83 | A/G | G/A | C/G | A/T | G/G | A/A | C/C | T/T | G/C | T/A |
| 84 | A/G | G/A | C/G | A/T | A/G | G/A | A/C | C/T | G/C | T/A |
| 85 | G/G | A/A | G/G | T/T | G/G | A/A | C/C | T/T | C/C | A/A |
| 86 | A/G | G/A | C/G | A/T | G/G | A/A | C/C | T/T | C/C | A/A |
| 87 | A/G | G/A | C/G | A/T | A/G | G/A | A/C | C/T | G/C | T/A |

North Indian population:

|  | **rs10009426** | **rs6824707** | **rs6851073** | **rs2271046** | **rs225160** | **rs225170** | **rs999634** | **rs3860707** | **rs35414474** | **rs17611952** |
| --- | --- | --- | --- | --- | --- | --- | --- | --- | --- | --- |
| 1 | A/G | G/A | C/G | A/T | A/G | G/A | A/C | C/T | C/C | A/A |
| 2 | A/G | G/A | C/G | A/T | G/G | A/A | C/C | T/T | G/G | T/T |
| 3 | A/G | G/A | C/G | A/T | A/G | G/A | A/C | C/T | C/C | A/A |
| 4 | A/G | G/A | C/G | A/T | A/G | G/A | A/C | C/T | C/C | A/A |
| 5 | A/G | G/A | C/G | A/T | A/A | G/A | A/C | C/T | C/C | A/A |
| 6 | A/G | G/A | C/G | A/T | A/G | G/A | A/C | C/C | C/C | A/A |
| 7 | A/A | G/G | C/C | A/A | A/G | G/A | A/C | C/T | C/C | A/A |
| 8 | A/A | G/G | C/C | A/A | A/A | G/G | A/A | C/C | C/C | A/A |
| 9 | A/G | G/A | C/G | A/T | G/G | A/A | C/C | T/T | G/G | T/T |
| 10 | G/G | A/A | G/G | T/T | G/G | A/A | C/C | C/T | C/C | A/A |
| 11 | G/G | A/A | G/G | T/T | G/G | A/A | C/C | T/T | G/C | T/A |
| 12 | A/A | G/G | C/C | A/A | A/A | G/G | A/A | C/C | C/C | A/A |
| 13 | A/G | G/A | C/G | A/T | A/G | G/A | A/C | T/T | G/C | T/A |
| 14 | G/G | A/A | G/G | T/T | G/G | A/A | C/C | C/T | G/C | T/A |
| 15 | G/G | A/A | G/G | T/T | G/G | A/A | C/C | T/T | C/C | A/A |
| 16 | A/G | G/A | C/G | A/T | A/G | G/A | A/C | C/T | C/C | A/A |
| 17 | A/G | G/A | C/G | A/T | A/G | G/A | A/C | C/T | C/C | A/A |
| 18 | A/G | G/A | C/G | A/T | G/G | A/A | C/C | T/T | G/C | T/A |
| 19 | A/A | G/G | C/C | A/A | A/G | G/A | A/C | C/T | G/C | T/A |
| 20 | G/G | A/A | G/G | T/T | G/G | A/A | C/C | C/T | C/C | A/A |
| 21 | A/G | G/A | C/G | A/T | A/G | G/A | C/C | T/T | G/C | T/A |
| 22 | G/G | A/A | G/G | T/T | G/G | A/A | C/C | T/T | G/C | T/A |
| 23 | G/G | A/A | G/G | T/T | G/G | A/A | C/C | C/T | C/C | A/A |
| 24 | A/A | G/G | C/C | A/A | A/G | G/A | A/C | T/T | G/C | T/A |
| 25 | A/G | G/A | C/G | A/T | A/G | G/A | A/C | C/T | C/C | A/A |
| 26 | A/A | G/G | C/C | A/A | A/G | G/A | A/C | C/T | G/C | T/A |
| 27 | G/G | A/A | G/G | T/T | G/G | A/A | C/C | T/T | C/C | A/A |
| 28 | G/G | A/A | G/G | T/T | G/G | A/A | C/C | T/T | C/C | A/A |
| 29 | A/G | G/A | C/G | A/T | G/G | A/A | C/C | T/T | G/G | T/T |
| 30 | G/G | A/A | G/G | T/T | G/G | A/A | C/C | T/T | C/C | A/A |
| 31 | A/A | G/G | C/C | A/A | A/G | G/A | A/C | C/T | C/C | A/A |
| 32 | A/G | G/A | C/G | A/T | G/G | A/A | C/C | T/T | G/C | T/A |
| 33 | A/G | G/A | C/G | A/T | A/G | G/A | A/C | C/T | C/C | A/A |
| 34 | A/A | G/G | C/C | A/A | G/G | A/A | C/C | T/T | G/G | T/T |
| 35 | A/G | G/A | C/G | A/T | G/G | A/A | C/C | T/T | G/C | T/A |
| 36 | G/G | A/A | G/G | T/T | G/G | A/A | C/C | C/T | C/C | A/A |
| 37 | G/G | A/A | G/G | T/T | G/G | A/A | C/C | T/T | C/C | A/A |
| 38 | A/G | G/A | C/G | A/T | A/G | G/A | A/C | C/T | G/C | T/A |
| 39 | A/A | G/G | C/C | A/A | A/G | G/A | A/C | C/T | G/C | T/A |
| 40 | A/G | G/A | C/G | A/T | A/G | G/A | A/A | C/C | C/C | A/A |
| 41 | A/A | G/G | C/C | A/A | A/A | G/G | A/A | C/C | C/C | A/A |
| 42 | A/G | G/A | C/G | A/T | A/G | G/A | A/C | C/T | C/C | A/A |
| 43 | A/G | G/A | C/G | A/T | A/G | G/A | A/C | C/T | G/C | T/A |
| 44 | G/G | A/A | G/G | T/T | G/G | A/A | A/C | C/T | C/C | A/A |
| 45 | A/G | G/A | C/G | A/T | A/G | G/A | A/C | C/T | G/C | T/A |
| 46 | A/G | G/A | C/G | A/T | A/G | G/A | A/C | C/T | C/C | A/A |
| 47 | A/G | G/A | C/G | A/T | A/G | G/A | C/C | T/T | C/C | A/A |
| 48 | G/G | A/A | G/G | T/T | G/G | A/A | C/C | T/T | C/C | A/A |
| 49 | A/A | G/G | C/C | A/A | A/G | G/A | A/C | C/T | G/C | T/A |
| 50 | A/G | G/A | C/G | A/T | A/G | G/A | A/C | C/T | G/C | T/A |
| 51 | A/A | G/G | C/C | A/A | A/A | G/G | A/A | C/T | C/C | A/A |
| 52 | G/G | A/A | G/G | T/T | G/G | A/A | C/C | T/T | C/C | A/A |
| 53 | A/A | G/G | C/C | A/A | A/A | G/G | A/A | C/C | C/C | A/A |
| 54 | G/G | A/A | G/G | T/T | G/G | A/A | A/C | C/T | C/C | A/A |
| 55 | A/A | G/G | C/C | A/A | A/A | G/G | A/A | C/C | C/C | A/A |
| 56 | A/G | G/A | C/G | A/T | A/G | G/A | A/C | C/T | G/C | T/A |
| 57 | A/G | G/A | C/G | A/T | G/G | A/A | C/C | C/T | G/C | T/A |
| 58 | A/G | G/A | C/G | A/T | A/G | G/A | A/C | C/T | C/C | A/A |
| 59 | G/G | A/A | G/G | T/T | G/G | A/A | C/C | C/T | G/C | T/A |
| 60 | A/G | G/A | C/G | A/T | G/G | A/A | C/C | T/T | G/G | T/T |
| 61 | A/A | G/G | C/C | A/A | G/G | G/A | A/C | C/T | C/C | A/A |
| 62 | G/G | A/A | C/G | T/T | A/G | G/A | C/C | T/T | C/C | A/A |
| 63 | A/A | G/G | C/C | A/A | A/A | G/G | A/A | C/C | C/C | A/A |

**S3 table:** The frequency of the haplotypes reconstructed from the SNPs rs10009426, rs6824707, rs2271046, rs35414474 and rs17611952 in the background of c. 544T>G mutation in cases, related controls and unrelated south and north Indian population.

| **Haplotypes** | **Frequency in cases (N=14)** | **Frequency in unrelated control north Indian population (N=63)** | **CHISQ** | **P-value** |
| --- | --- | --- | --- | --- |
| H1' | 0.9 | 0.1251 | 37.1 | 1.12E-09 |
| H2' | 0 | 0.2996 | 4.138 | 0.04194 |
| H3' | 0 | 0.2964 | 4.079 | 0.04342 |
| H4' | 0 | 0.06626 | 0.7097 | 0.3996 |
| H5' | 0 | 0.02755 | 0.2819 | 0.5955 |
| H6' | 0 | 0.02561 | 0.2658 | 0.6061 |
| H7' | 0 | 0.02439 | 0.2495 | 0.6174 |
| H8' | 0 | 0.01626 | 0.1651 | 0.6845 |
| H9' | 0.1 | 0.1188 | 0.03176 | 0.8586 |
| **Haplotypes** | **Frequency in related controls (N=14)** | **Frequency in unrelated control north Indian population (N=63)** | **CHISQ** | **P-value** |
| H1' | 0.5652 | 0.1251 | 25.2 | 5.17E-07 |
| H2' | 0.12 | 0.2996 | 3.406 | 0.06496 |
| H3' | 0.2348 | 0.2964 | 0.3863 | 0.5343 |
| H4' | 0 | 0.06626 | 1.753 | 0.1855 |
| H5' | 0.005183 | 0.02755 | 0.4479 | 0.5033 |
| H6' | 0 | 0.02561 | 0.6541 | 0.4186 |
| H7' | 0 | 0.02439 | 0.6224 | 0.4302 |
| H8' | 0 | 0.01626 | 0.4121 | 0.5209 |
| H9' | 0.07482 | 0.1188 | 0.406 | 0.524 |
| **Haplotypes** | **Frequency in cases (N=14)** | **Frequency in unrelated control south Indian population (N=87)** | **CHISQ** | **P-value** |
| H1' | 0.9 | 0.1463 | 35.18 | 3.00E-09 |
| H2' | 0 | 0.2505 | 3.281 | 0.07007 |
| H3' | 0 | 0.373 | 5.759 | 0.0164 |
| H4' | 0 | 0.01146 | 0.1159 | 0.7335 |
| H5' | 0 | 0.02251 | 0.2263 | 0.6343 |
| H7' | 0 | 0.01232 | 0.1235 | 0.7252 |
| H8' | 0 | 0.0138 | 0.137 | 0.7113 |
| H9' | 0.1 | 0.1135 | 0.01449 | 0.9042 |
| H10' | 0 | 0.03521 | 0.3599 | 0.5486 |
| H11' | 0 | 0.02403 | 0.2433 | 0.6218 |
| **Haplotypes** | **Frequency in related controls (N=14)** | **Frequency in unrelated control south Indian population (N=87)** | **CHISQ** | **P-value** |
| H1' | 0.5652 | 0.1463 | 23.37 | 1.34E-06 |
| H2' | 0.12 | 0.2505 | 2.148 | 0.1427 |
| H3' | 0.2348 | 0.373 | 1.876 | 0.1707 |
| H5' | 0.005183 | 0.02251 | 0.3887 | 0.533 |
| H7' | 0 | 0.01232 | 0.3113 | 0.5769 |
| H8' | 0 | 0.0138 | 0.3493 | 0.5545 |
| H9' | 0.07482 | 0.1135 | 0.3124 | 0.5762 |
| H10' | 0 | 0.03521 | 0.9082 | 0.3406 |
| H11' | 0 | 0.02403 | 0.6136 | 0.4334 |

**S4 table:** Proportion of loci shares to be zero alleles (Z0), one allele (Z1), or two alleles (Z=2) and PI_HAT values between individual pairs of the same family pedigree.

| **Individual ID1** | **Individual ID2** | **Z0** | **Z1** | **Z2** | **PI_HAT** |
| --- | --- | --- | --- | --- | --- |
| SG-3-S | SG-3-P1 | 0.0365 | 0.8178 | 0.1457 | 0.5546 |
| SG-3-S | SG-3 | 0.0462 | 0.8522 | 0.1016 | 0.5277 |
| SG-3-P1 | SG-3 | 0.2554 | 0.4159 | 0.3287 | 0.5366 |
| SG-4-P1 | SG-4-P2 | 0.8951 | 0.049 | 0.056 | 0.0804 |
| SG-4-P1 | SG-4 | 0.0401 | 0.8628 | 0.097 | 0.5284 |
| SG-4-P2 | SG-4 | 0.0401 | 0.8805 | 0.0794 | 0.5196 |
| SG-6-P1 | SG-6-P2 | 0.7309 | 0.1414 | 0.1277 | 0.1984 |
| SG-6-P1 | SG-6 | 0.1046 | 0.6902 | 0.2052 | 0.5503 |
| SG-6-P2 | SG-6 | 0.0936 | 0.697 | 0.2094 | 0.5579 |
| SG-7-P1 | SG-7 | 0.062 | 0.7099 | 0.228 | 0.583 |
| SG-8-P1 | SG-8-P2 | 0.8748 | 0 | 0.1252 | 0.1252 |
| SG-8-P1 | SG-8 | 0.0632 | 0.8589 | 0.0779 | 0.5073 |
| SG-8-P2 | SG-8 | 0.0535 | 0.8483 | 0.0982 | 0.5223 |
| SG-9-P1 | SG-9-P2 | 1 | 0 | 0 | 0 |
| SG-9-P1 | SG-9 | 0.0426 | 0.8897 | 0.0677 | 0.5126 |
| SG-9-P2 | SG-9 | 0.0401 | 0.8929 | 0.067 | 0.5134 |
| SG-10-P1 | SG-10-P2 | 0.9036 | 0.042 | 0.0544 | 0.0754 |
| SG-10-P1 | SG-10 | 0.0693 | 0.8734 | 0.0573 | 0.494 |
| SG-10-P2 | SG-10 | 0.0645 | 0.7995 | 0.1361 | 0.5358 |
| SG-12-P1 | SG-12 | 0.0511 | 0.8063 | 0.1427 | 0.5458 |

**S5 table:** Proportion of loci shares to be zero alleles (Z0), one allele (Z1), or two alleles (Z=2) and PI_HAT values between seemingly unrelated 33 individual pairs from different pedigrees sharing a novel relationship with Z1 and Z2 IBD share.

| **Family ID1** | **Individual ID1** | **Family ID2** | **Individual ID2** | **Z0** | **Z1** | **Z2** | **PI_HAT** |
| --- | --- | --- | --- | --- | --- | --- | --- |
| Ped2 | SG-2 | Ped10 | SG-10-P2 | 0.9011 | 0 | 0.0989 | 0.0989 |
| Ped3 | SG-3-S | Ped4 | SG-4-P1 | 0.9298 | 0 | 0.0702 | 0.0702 |
| Ped3 | SG-3-S | Ped4 | SG-4-P2 | 0.9604 | 0 | 0.0396 | 0.0396 |
| Ped3 | SG-3-S | Ped4 | SG-4 | 0.9494 | 0 | 0.0506 | 0.0506 |
| Ped3 | SG-3-S | Ped8 | SG-8-P1 | 0.9486 | 0.0092 | 0.0422 | 0.0468 |
| Ped3 | SG-3-S | Ped9 | SG-9 | 0.945 | 0 | 0.055 | 0.055 |
| Ped3 | SG-3-S | Ped10 | SG-10-P1 | 0.9304 | 0.0335 | 0.0361 | 0.0529 |
| Ped3 | SG-3-S | Ped10 | SG-10-P2 | 0.9243 | 0.0108 | 0.065 | 0.0703 |
| Ped3 | SG-3-S | Ped12 | SG-12-P1 | 0.8708 | 0.1085 | 0.0207 | 0.075 |
| Ped4 | SG-4-P1 | Ped10 | SG-10-P2 | 0.9421 | 0 | 0.0579 | 0.0579 |
| Ped4 | SG-4-P1 | Ped12 | SG-12-P1 | 0.9231 | 0.043 | 0.0339 | 0.0554 |
| Ped4 | SG-4-P2 | Ped6 | SG-6-P2 | 0.932 | 0 | 0.068 | 0.068 |
| Ped4 | SG-4-P2 | Ped10 | SG-10-P1 | 0.9425 | 0.0218 | 0.0357 | 0.0466 |
| Ped4 | SG-4-P2 | Ped10 | SG-10 | 0.953 | 0 | 0.047 | 0.047 |
| Ped4 | SG-4-P2 | Ped12 | SG-12-P1 | 0.8987 | 0.0665 | 0.0348 | 0.068 |
| Ped4 | SG-4 | Ped6 | SG-6-P2 | 0.9166 | 0 | 0.0834 | 0.0834 |
| Ped4 | SG-4 | Ped10 | SG-10-P1 | 0.9492 | 0 | 0.0508 | 0.0508 |
| Ped4 | SG-4 | Ped10 | SG-10-P2 | 0.9373 | 0 | 0.0627 | 0.0627 |
| Ped4 | SG-4 | Ped12 | SG-12-P1 | 0.889 | 0.0691 | 0.0419 | 0.0764 |
| Ped6 | SG-6-P2 | Ped10 | SG-10-P1 | 0.9259 | 0 | 0.0741 | 0.0741 |
| Ped6 | SG-6-P2 | Ped10 | SG-10-P2 | 0.9133 | 0.0348 | 0.0519 | 0.0693 |
| Ped6 | SG-6-P2 | Ped10 | SG-10 | 0.924 | 0 | 0.076 | 0.076 |
| Ped6 | SG-6-P2 | Ped12 | SG-12-P1 | 0.9245 | 0 | 0.0755 | 0.0755 |
| Ped8 | SG-8-P1 | Ped10 | SG-10-P2 | 0.9548 | 0 | 0.0452 | 0.0452 |
| Ped8 | SG-8-P1 | Ped12 | SG-12-P1 | 0.9583 | 0.0011 | 0.0406 | 0.0411 |
| Ped8 | SG-8-P2 | Ped10 | SG-10-P2 | 0.9106 | 0 | 0.0894 | 0.0894 |
| Ped8 | SG-8-P2 | Ped12 | SG-12-P1 | 0.9274 | 0 | 0.0726 | 0.0726 |
| Ped8 | SG-8 | Ped10 | SG-10-P2 | 0.9229 | 0 | 0.0771 | 0.0771 |
| Ped8 | SG-8 | Ped12 | SG-12-P1 | 0.9583 | 0.0072 | 0.0345 | 0.0381 |
| Ped9 | SG-9-P2 | Ped12 | SG-12-P1 | 0.9612 | 0 | 0.0388 | 0.0388 |
| Ped10 | SG-10-P1 | Ped12 | SG-12-P1 | 0.8951 | 0.0611 | 0.0438 | 0.0743 |
| Ped10 | SG-10-P2 | Ped12 | SG-12-P1 | 0.8902 | 0.0473 | 0.0624 | 0.0861 |
| Ped10 | SG-10 | Ped12 | SG-12-P1 | 0.9121 | 0.0523 | 0.0356 | 0.0617 |
